# Selection for Molecularly Complementary Modules (MCMs) Drives the Origins and Evolution of Pleiofunctional, Epistatic Interactomes (PEIs)

**DOI:** 10.3390/life16010170

**Published:** 2026-01-20

**Authors:** Robert Root-Bernstein

**Affiliations:** Department of Physiology, Michigan State University, East Lansing, MI 48824, USA; rootbern@msu.edu

**Keywords:** origins, interactome, pleiofunctional, epistatic, molecular complementarity

## Abstract

The huge number of possible permutations of genes, proteins and small molecules make the random emergence of cellular networks problematic. How, therefore, do interactomes come into existence? What selects for their stability and functionality? I hypothesize that interactomes originate from molecularly complementary modules (MCMs) that are selected for stability and retain their interactivity when mixed and matched with other such modules to create novel molecules and complexes displaying emergent properties not present in the individual components of the network. Because evolution can only proceed by working upon existing variants, and these variants emerge from selection of MCMs, the resulting systems must exhibit the characteristics of pleiofunctional, epistatic interactomes (PEIs). The resulting systems should display “molecular paleontology”, providing clues as to the historical process by which these MCMs were incorporated into the system. The MCM mechanism of PEI evolution is illustrated here by two case studies. The first concerns the prebiotic emergence of the glutathione–ascorbate anti-oxidant system and its later incorporation into regulation of glucose transport and catecholamine receptor activity. The second concerns the MCM evolution of the ribosome as, perhaps, the first PEI, and its role as a module for the later construction of the first cellular genomes.

## 1. Introduction: The Problem of Explaining How Pleiofunctional, Epistatic Interactomes (PEI) Evolved to Characterize Living Systems

François Jacob argued that evolution is a tinkerer, making use of whatever materials are available to cobble together new components through a process that he called “bricolage” rather than inventing new ones from scratch [1]. The result, he argued, is that every molecular system is the result of previous historical selection processes. This process leaves what might be called a “molecular paleontology” capturing traces of its ontogeny. Jacob also argued that evolution selected not for the fitness of individual components, but rather for integrated regulatory circuits, changes in which drive evolutionary novelties [2]. The major question that evolutionary theory must therefore address is how, in the face of random mutations that should result in the diversification or disintegration of integrated regulatory circuits, erasing the historical path by which these circuits evolved, extremely robust, persistent interactomes have not only evolved, but dominated cellular structures and functions.

Jacob’s contention that evolution is a tinkerer of integrated regulatory circuits is almost certainly correct. Three of the most interesting characteristics of cellular systems are that most, if not all, of the molecular components are pleiofunctional; epistatic control predominates; and interactomes govern both genetic and metabolic control of functions. These three phenomena pose a challenge to the standard story presented by the modern evolutionary synthesis, which is based on selection acting upon divergent genetic, epigenetic and developmental variants. While this modern synthesis is useful for explaining the diversity, distributions and adaptations of life forms, it does not address how evolution produces increasingly integrated systems that are so constrained that both their genetics and metabolism are highly conserved across phyla. My thesis is that selection for molecularly complementary modules counteracts and seriously constrains the diversification introduced by random variations, giving rise to living systems characterized by highly conserved, pleiofunctional, epistatic interactomes.

The pleiofunctionality of molecules employed by cellular systems is a fairly recent observation. An early misconception common to both genetics and biochemistry was that each molecule in a living system had one, primary role and was involved in the expression of one trait. This idea sometimes persists even today and is evidenced by the fact that in most textbooks and many research papers, biomolecules tend to be described as having one particular function. In fact, most, if not all, are pleiofunctional, meaning that they are not limited to one specific cellular task but contribute in diverse ways to multiple processes or systems and therefore affect multiple traits. Adenosine triphosphate (ATP) is a good example. ATP provides chemical energy to drive many types of reactions [3]; acts as a building block of polynucleotides [3]; mediates signal transduction for many classes of receptors [3]; catalyzes tau protein aggregation [4]; inhibits neurodegeneration-associated fibrilization [5]; binds to RNA recognition motifs (RRM) on a very wide range of proteins, regulating their activity [6]; dissociates actin–myosin complexes [3]; and is co-stored and co-released with neurotransmitters such as acetylcholine, noradrenaline, and glutamate in both the central and peripheral nervous systems [7]. So, one problem that evolutionary theory needs to address is how molecules acquire multiple functions.

Epistatic control of genetic and metabolic pathways is another characteristic of living systems that is taking on increasingly important evolutionary implications. Epistasis is a phenomenon in which the expression of one gene is modified by one or more other genes. While Mendelian genetics was based upon the assumption that each gene regulated a single trait (i.e., one protein) that had one function, even Mendel was aware that his “laws” applied only to exceptional cases [8]. In reality, nearly all genes are regulated by promotors and repressors that control multiple genes; feedback and feedforward regulation is rarely limited to a single gene; and gene products are invariably pleiofunctional. Thus, the vast majority of traits are under epistatic regulation so that their expression is a result of the interactions of several or many genetic and metabolic pathways (e.g., [9,10,11]).

Within the context of evolutionary change, epistasis has particularly been implicated in long-term evolutionary experiments as a key result of selection for increased fitness. Lenski’s studies of *Escherichia coli* (*E. coli*) adaptations under nutrient limitations over tens of thousands of generations documented coordinated mutations in epistatically regulated genes that governed DNA superhelicity and the stringent response [12,13]. These two responses are themselves interconnected systems. These studies also observed the evolution of a new trait, the use of citrate as an energy source, as a result of pairing specific mutations in a dicarboxylic antiporter with a carboxylic acid transporter to create a new network [14,15,16,17]. An independent study of the emergence of citrate usage in multiple *E. coli* populations also observed selection for epistatic control [18]. Modeling of the long-term *E. coli* experiments have universally concluded that selection for increased fitness is invariably accompanied by novel forms of epistatic regulation [19,20,21,22,23,24,25,26,27]. Other long-term evolutionary studies of *E. coli* under types of selection other than limited nutrients, such as antibiotic exposure [28,29], as well as long-term evolutionary studies of other organisms under a variety of selection pressures [30,31,32], have also implicated increased epistasis as a mechanism driving increased fitness. Accounting for selection for ever-increasing epistasis is therefore an important challenge for evolutionary theory. How is epistatic networking achieved?

This question leads naturally to the observation that all living systems are characterized by highly integrated interactomes. Interactomes are defined as the entire set of interactions between the constituents of a system such as a cell [33] (Sanchez, et al., 1999). One may also speak of interactomes of different types depending on the hierarchy being observed, whether organelle, cellular, organismal or ecological, or depending on the types of molecules being considered. Interactomes may be composed of gene networks, protein interactions, lipids, small molecule metabolites, or any combination of these. Additionally, interactomes characterize all types of living systems from viruses through bacteria to yeasts and human beings. As far as I have been able to determine by examining the myriad biochemical pathways illustrated in textbooks, randomly dipping into SwissProt protein database pages (which list known protein interaction data) and a multi-decade career as a physiologist and drug developer with broad experience of many metabolic systems, there is no molecular constituent of any living system that is not a part of an interactome. This conclusion is, of course, subject to falsification, but for the moment, working on the proposition that no gene, no protein and no metabolite functions independently of interactions with (at least) several other molecular constituents poses an important evolutionary puzzle in need of explanation.

Surprisingly, no general evolutionary mechanism has been proposed that can account for pleiofunctional, epistatic interactome (PEI) formation. While there is a robust body of literature on the ways in which pre-existing interactomes can adapt through selection on variants (e.g., [34,35,36,37,38,39,40]), I have been unable (with the few exceptions to be described below) to identify any articles or books that describe the origins of interactomes de novo, nor have reviewers of this paper brought any to my attention. Indeed, Tompa and Rose [41] have pointed out that the emergence of interactomes is far from being an obvious or predetermined characteristic of living systems because the number of possible permutations of chemical constituents available prebiotically (or even within the more limited chemical ecology of a cell) is literally astronomical (the so-called “Levinthal paradox of interactomes”). This is not to say that research on the origins of interactomes does not exist, but at best the literature is very sparse and indicates that the origins problem is one that has largely been ignored or overlooked. This paper develops general theoretical considerations that suggest prebiotic and early biotic selection for molecularly complementarity modules (MCMs) may have played a significant role in providing a mechanism for interactome origins, a general theoretical approach also explored by Caetano-Anollés et al. [42] and Csermely [43]. This idea was first proposed by Root-Bernstein and Dillon in 1997 [44] and developed more generally in subsequent papers [45,46,47,48,49], but has only recently been developed through the synthesis of experiment- and observation-based specific examples of receptor and transporter evolution in a forthcoming book chapter [50]. The present paper extends the MCM mechanism to additional metabolic systems. Whether MCM is the major driver of PEI formation cannot, of course, be demonstrated irrefutably based on only a handful of case studies. Many additional interactomes must be investigated and the possibility that there are other mechanisms by which PEIs originate must be considered. My purpose here is to establish nothing more than the plausibility of the MCM mechanism and to model the types of research and evidence that may be needed to extend the idea.

## 2. Some Previously Characterized MCM Systems Producing PEI

The basic hypothesis proposed here is that selection for modern cellular systems began within the prebiotic chemical milieu where molecularly complementary molecules (MCMs) were more likely to survive various degradative processes and thus to become the materials upon which further adaptations could evolve. These surviving modules were then mixed and matched to produce variations that were necessarily networked with each other because of the complementarity inherent in the interactive component modules from which the larger molecules were derived.

One explicit assumption of my hypothesis is that nature does not explore every possible variation of the building blocks of life, nor does it generate every possible permutation of them, but rather nature is, as Jacob suggested, a “cobbler” that works with whatever is most abundant and/or stable and/or functional at any given time during the evolutionary process. Thus, MCMs not only provide a means of networking the novel constructs that nature cobbles together but also set extraordinary constraints on the possible variants that it explores.

Various testable implications follow from the MCM hypothesis for explaining PEI. The most important and general are that molecules that bind to each other are likely to have been harnessed to regulate each other’s cellular or physiological activity and, conversely, that when molecules are found to alter each other’s cellular or physiological activity, they will most likely be molecularly complementary [44,51]. Thus, molecular interactions should be predictors of MCMs and MCMs should be predictors of interactive functionality. Both versions of the relationship result directly from evolutionary selection for PEI.

Many specific and testable implications follow. One concerns the particular evolution of receptors and transporters from molecularly complementary modules. Functional cells require receptor–ligand pairs, so how did the first receptors and ligands evolve and become functionally integrated? If the receptors and ligands evolved separately from random variations in protein sequences and peptides or other small molecular variants, what selection process paired any given ligand with any given receptor and how was the resulting complex linked to a particular function? Given the huge number of possible receptor sequences (the permutations of a 400 amino acid protein with any of the 20 amino acids at any position runs to about 1/10^600^) and at least 170 billion small molecules to choose from [52], not to mention the equally large number of possible peptide ligands that evolution could produce, the number of possible combinations is literally astronomical [41], a clear example of the Levinthal paradox of interactome formation. Yet even the earliest cells would have needed receptors and transporters in order to maintain their integrity and metabolism. So, the solution to these questions must involve some process that drastically limits the permutations so as to link one receptor sequence with one ligand and do so functionally [50,53,54].

Dwyer [55] proposed a clever solution to these puzzles based on MCMs. He hypothesized that peptide receptors evolved from self-complementary peptides through a process in which one element of the pair became the ligand and the other a protoreceptor ligated to one or more transmembrane spanning modules [55]. As the receptor was subsequently elaborated to interact with second messengers and to be amenable to allosteric control, the original protoreceptor sequence would have been conserved at the ligand binding site.

A testable implication of Dwyer’s hypothesis was that some peptide receptors should have copies of their ligand within the receptor binding site. Dwyer demonstrated, for example, that bungarotoxin, an acetylcholine (ACh)-binding peptide, binds to a region of the ACh receptor that mimics the toxin’s sequence. That receptor sequence also binds ACh. Similar self-similarity was found for alpha-scorpion toxins and the sodium channel sequences to which they bind and for interleukin-2 and its receptor and [55]. Dwyer went on to demonstrate that modules or domains such as that responsible for Il-2 binding to its receptor are highly conserved among related gastrointestinal peptides with which Il-2 interacts physiologically [56], leading him to produce evidence that networking is implemented by means of highly conserved transposable exons, or “trexons”, capable of integration into other gene sequences to create novel protein sequences with similar binding affinities [57,58]. In this way, Dwyer’s theory integrates complementary modularity with the emergence of epistatic control.

I extended Dwyer’s theory beyond self-aggregating peptides and their mimics to any (hetero)complementary pair of molecules in which one of the pair is a peptide. The peptide becomes the core of the evolving receptor’s ligand binding site and the particular pairing confers specific functionality on the ligand [53,54]. The evolution of opioid peptide receptors and their structural and functional relationships to aminergic receptors, as well as their enhancement by aminergic compounds, provide good examples.

To begin with opioid peptides self-aggregate and are therefore homo-complementary (reviewed in [59]). It follows from Dwyer’s theory that opioid peptide-like sequences should be found in the opioid binding sites of opioid receptors. This is the case; proenkephalin- and endorphin-like sequences are present in opioid receptor binding sites and peptides derived from these sequences have been shown to have significant affinity for opioid peptides [59].

**Figure 1:**
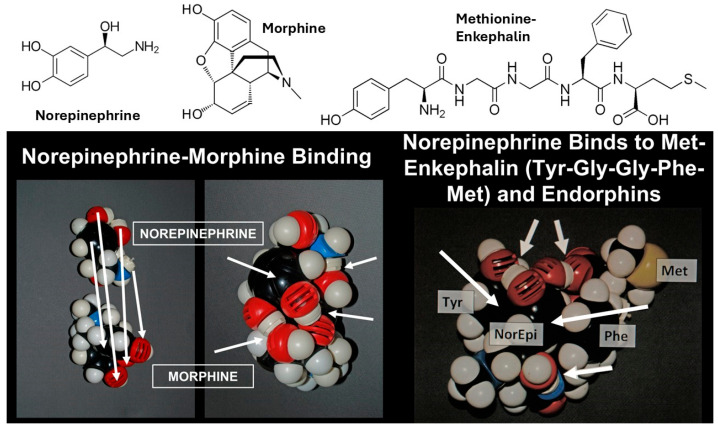
CPK models demonstrating binding of norepinephrine to morphine and Met-enkephalin. Arrows show where bonds are being formed. These include pi–pi overlap bonds between the ring structures and hydrogen or ionic bonds between hydroxyls and/or amines. Oxygens are red; nitrogens, blue; carbons, black; hydrogens, white; sulfur, yellow. The folded structure of Met-enkephalin that forms a pocket for binding norepinephrine that is utilized in the model is based on nuclear magnetic resonance studies by Sanfelice and Temussi [68] and studies of Leu-enkephalin by Amodeo, et al. [69]. This folded structure forms a pocket that is presumably stabilized by the binding of catecholamines into it. The similarity in essential structural features of morphine and the enkephalins has previously been described by Wu and Hruby [70] and Root-Bernstein and Dillon [71], which helps to explain their mutual complementarity to catecholamines.

Additionally, we have demonstrated that aminergic compounds bind directly to opioids, including morphine, enkephalins and endorphins (Figure 1), resulting in very stable complexes resistant to degradation [60,61]. This mutual protection would have acted as a positive selection factor that may help to explain why opioids and adrenergic compounds are almost invariably synthesized, co-stored and co-released in neurons and the adrenal cortex [62,63,64,65,66,67].

Opioids and catecholamines are further known to alter each other’s pharmacological effects, often resulting in significant enhancement of the activity of both (e.g., [72,73]; reviewed in [74]). The mechanism for this enhancement is a result of endorphin-like modules found in aminergic receptor binding sites [75,76]; opioid binding to the adrenergic receptor at an extracellular allosteric site [71]; as well as heterodimerization of opioid and adrenergic receptors, providing for additional allosteric receptor control as a result of shared, complementary transmembrane modules [77,78,79]. This, and other, evidence suggests that adrenergic and opioid receptors, though often categorized as different classes of receptors, may have evolved through shared modularity [59,80]. Thus, conserved opioid–adrenergic complexes were selected as modules for use in the co-storage and release of these compounds and, as a result of swapping these modules to create novel sequences, as the basis for the evolution of adrenergic and opioid receptors with allosteric enhancement and metabolic networking—an example of how pleiofunctionality may arise (see [50] for a more detailed examination of this system).

Similarly, glucose regulation has been integrated by means of several peptides that have acted as complementary modules mediating opposed physiological functions. The most important of these are insulin and glucagon, which are not only physiologically complementary but also molecularly complementary [81]. The binding of these two peptides is so tight that they resist separation even through repeated crystallizations (indeed, glucagon was first identified as a contaminant of crystallized insulin and was present in all insulin preparations until it became possible to produce insulin by recombinant genetic means) [82]. In addition, both molecules self-aggregate, insulin into dimers and hexamers [83] and glucagon into dimers [84]. As predicted by Dwyer’s theory, the binding regions of the insulin receptor exhibit multiple insulin-like sequences while the binding site of the glucagon receptor exhibits multiple glucagon-like sequences [53,54,85,86]. Following my extension of Dwyer’s theory to hetero-complementary molecules, each receptor binding site also has a couple of sequences mimicking the complementary peptide [53,54,85,86]. The exons for insulin and glucagon mimic regions of the receptor exons, thereby satisfying Dwyer’s suggestion that these may have originated as trexons.

**Figure 2:**
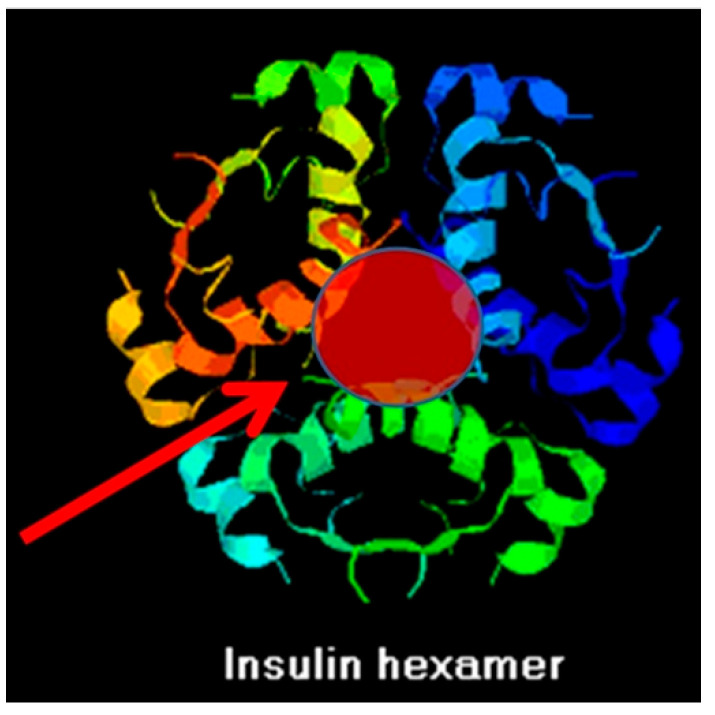
Insulin, which binds glucose, self-aggregates into amphipathic hexamers capable of inserting into lipid membranes. These hexamers have a pore (red dot) of appropriate size to transport glucose (based on [88]).

**Figure 3:**
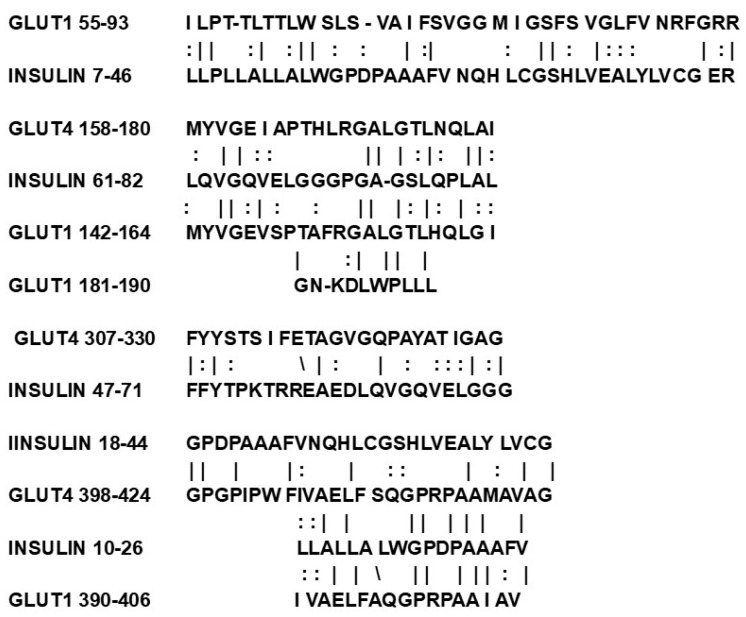
Some of the significant similarities between human insulin (which has multiple binding sites for glucose) and human glucose transporters 1 and 4. All of these (and additional similarities) are found solely in transmembrane sequences that form the glucose transport core. These similarities suggest that the gene for insulin may have been a module duplicated for the emergence of the transporters [88].

Glucose transporters may also have evolved from insulin-like modules. The physiological function of insulin is to permit blood glucose to enter cells. Notably, insulin directly binds glucose at two high-affinity and four low-affinity sites [87]; and all glucose transporters have multiple insulin-like sequences that occur specifically in the regions forming the glucose transport core (Figure 2) [88]. Indeed, insulin self-aggregates into hexamers (Figure 3) that notably have an interior hole of appropriate size for glucose to fit. Thus, ligating several insulin genes could have provided the basis for evolving a glucose transporter [88].

Thus, an insulin-like module appears to have been integrated into its own receptor function as well as into glucagon receptor function and also provided a building block for the evolution of glucose transport. Glucose–insulin–glucagon complementarity therefore lies at the heart of networking key elements of glucose regulation. Moreover, this networking extends to the genetic control of glucose metabolism more generally: insulin and glucagon have opposing actions through second messenger systems on the expression of all the genes in the glycolytic and gluconeogenic pathways, which can themselves be viewed as an interactome consisting of a series of three opposing substrate cycles [89,90]. Moreover, insulin receptor and insulin-like growth factor receptors are known to translocate from the cell membrane into the nucleus, where they bind directly to their own gene promoters as a further regulatory system [91], thus demonstrating molecular complementarity to a section of their own DNA. (A more complete examination of this modular system is provided in [50]).

In sum, opioid, adrenergic, insulin and glucagon receptor evolution, as well as glucose transport, appear to have been constrained by MCMs resulting in PEIs [50].

## 3. PEI Evolution Case 1: Ascorbic Acid, Glutathione, Glucose Transporters and Aminergic Receptors

Further examples of MCMs constraining the emergence of PEIs have also been characterized and two of these are reviewed here. The first concerns the possible origins of a pleiofunctional ascorbic acid (vitamin C) interactome.

While the importance of modularity in the evolution of protein sequences and structures has been widely explored for many decades [42,92,93,94,95], and is evident in the two examples just provided above, the emergence of modularity more broadly within an evolutionary context, and especially as it regards small molecule complementarity, has received much less research and is particularly absent in discussions of the emergence of networking [96,97]. My first extended example of how MCM has been implemented in networking a metabolic system therefore focuses on an unusual case involving the small molecules of ascorbic acid, glutathione and catecholamines and ways in which their interactions have been integrated into aminergic receptor functions and glucose transport and regulation.

Ascorbic acid has been identified as a very early product of prebiotic chemistry, the presence of which contributed hundreds-fold to increased synthesis of amino acids [98,99]. It is therefore very likely that processes that fostered ascorbic acid synthesis and recycling would have been highly adaptive, improving the fitness of the prebiotic pre-protein world. One likely candidate for fostering ascorbic acid synthesis and preservation is the tripeptide glutathione, which, in modern cellular systems binds to the oxidation product of ascorbic acid, dehydroascorbic acid, catalyzing its transformation back into ascorbic acid [100].

Glutathione is notable in a prebiotic chemistry context for two properties. One is that it is one of the smallest biologically active peptides, consisting of a mere three amino acids: glutamyl–cysteinyl–glycine. Its constituent amino acids have been found in Miller-like experiments [98,101,102,103] so that its emergence just by random amino acid ligation would have been very likely and its stability and function selected for by binding to ascorbate. Secondly, glutathione is not genetically encoded or derived from a genetically encoded protein precursor by means of ribosomal translation of an mRNA. This suggests that glutathione emerged prior to genetic replication and translation. Instead, glutathione is synthesized de novo from its amino acid precursors by a pair of non-ribosomal peptide synthetase (NRPS) enzymes. The first produces glutamyl-cysteine, with the glycine being added afterwards [104]. While in modern cellular systems, this synthesis is optimized by a pair of enzymes, it must be remembered that enzymes simply optimize reactions by lowering energy barriers but do not enable reactions to occur that would not occur naturally. It is reasonable, therefore, to assume that since glutamic acid, cysteine and glycine were available prebiotically, glutathione would have been generated as well. In consequence, a primitive antioxidant recycling system would likely have been selected for increasing the “fitness” of the early prebiotic chemical environment by increasing amino acid synthesis, which in turn would have promoted synthesis of glutathione, optimizing ascorbate utilization and thereby creating a very simple feedforward cycle. Subsequently, peptides (and eventually proteins) that catalyzed the synthesis of glutathione [104] would also have been selected, further enhancing this cycle. The centrality of ascorbic acid utilization in cells may therefore have its origins in the earliest prebiotic chemistry.

**Figure 4:**
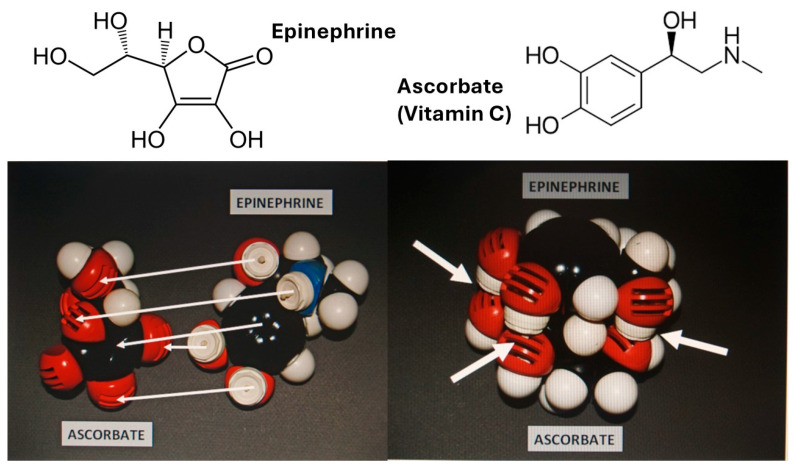
CPK models of epinephrine binding to ascorbate (vitamin C). Arrows indicate where bonds form. These include pi–pi overlap bonds between the ring structures and hydrogen or ionic bonds between hydroxyls and/or amines. Oxygens are red; nitrogens, blue; carbons, black; hydrogens, white. Binding based on data from [113,114,115].

Ascorbic acid has functions in addition to serving as an antioxidant and some of these functions are mediated by glutathione-like modules incorporated into other proteins. To begin with, ascorbic acid has many roles as an antioxidant, including the prevention of oxidation of adrenergic and histaminergic compounds [61,105,106,107,108]. Tartaric acid, which shares a structure similar to ascorbate [71], also acts as a potent antioxidant for catecholamines [109,110,111,112] and has similar binding properties [113,114]. However, its protection of catecholamines and histamines is implemented by means of an unusual mechanism. Rather than protecting these compounds from oxidation by oxidizing more rapidly than they do, ascorbic acid protects them through molecular complementarity. Ascorbate and cyclic amines form complexes by means of hydrogen and ionic bonds augmented by charge transfer complexation, which ties up all of the oxidizable residues on both the aminergic compound and the ascorbic acid itself (Figure 4) [113,115]. In consequence, these complexes are stable for many hours or days, while the individual compounds have half-lives of about 30 min at room temperature in a normal oxygen atmosphere [44,105,106,107,108,115,116,117]. The importance of the formation of such stable complexes in terms of selection for chemical fitness in a prebiotic system should be evident [41,43,45,48].

Evolution apparently took advantage of the stability of ascorbic acid–adrenergic compound complexes in multiple ways. One was to co-store these compounds in the same vesicles in the same cell types. These cells include neurons and the adrenal gland [118]. This mechanism has two benefits. One is to protect both compounds from degradation. Another is to couple the reduction of ascorbate to capture electron flow through the enzymes involved in catecholamine synthesis [119]. Equally importantly, by forming a complex, both molecules are effectively pulled out of the solution (precipitated) so that they can efficiently be stored in granules at very high concentrations and maintained against a concentration gradient [120] without energy input [121]. A fourth benefit of co-storage is that when adrenergic catecholamines such as epinephrine, norepinephrine and dopamine are released into neuronal synapses [122], or from the adrenals [123] into the blood stream (where, notably, the concentration of ascorbic acid is already between 50 and 75 µmol/L or about 0.9 to 1.3 mg/dL), the majority of the adrenergic compounds remain complexed to the ascorbic acid, thereby protecting both against degradative processes. Thus, more of the adrenergic compound reaches its target than would otherwise be the case. So, simple prebiotic chemistry plays a significant role in cellular and organismal physiology.

However, that is only the beginning of the ways that evolution has made use of ascorbate–aminergic compound complexing. Another has been to utilize the components to optimize adrenergic and histaminergic receptor activity. The adrenergic receptors discussed above are enhanced not only by opioids and opioid antagonists, but also by ascorbic acid [116,124]. A similar enhancement has been found in histaminergic receptors [117] and tartaric acid was found to be able to replace ascorbic acid as an effective catecholamine activity enhancer [114]. The mechanism of this enhancement has been localized to a pair of extracellular loops on these aminergic receptors that not only mimicked glutathione in sequence but also mimicked glutathione in recycling dehydroascorbic acid back into ascorbic acid [125,126]. Thus, the incorporation of glutathione-like modules into the extracellular loops of aminergic receptors took advantage of the co-storage and co-transmission of aminergic compounds to ensure their delivery to their receptors without becoming oxidized and then optimized catecholamine activity by means of allosteric enhancement of the ligand by its complement. Additionally, the receptor then recycles any dehydroascorbate that forms back into ascorbate.

In light of catecholamine–ascorbate interactions and the summary of catecholamine–opiate complementarity summarized above, a logical inference is that the ascorbate and opioid systems might interact as well and, in particular, that ascorbate and opiates might share some functions. That is, indeed, the case. Both ascorbate and opiates enhance aminergic receptor function [71]. However, since ascorbate lacks the multicyclic structure of opioids, it does not directly activate opioid receptors [75,76,125] but it can either synergize with opiates when co-administered [127,128] or antagonize when administered prior to the opiate [129,130]. Ascorbate can also treat some of the withdrawal symptoms associated with opioid addiction by acting directly as a weak analgesic [127] and by replacing the enhancement function that opioids play on the adrenergic system [131,132,133]. Thus, once again, complex physiological processes such as addiction and withdrawal appear to have their evolutionary basis in small molecule complementarity implemented through the incorporation of the associated modules into more complex protein networks and resulting in a variety of allosteric enhancement mechanisms.

**Figure 5:**
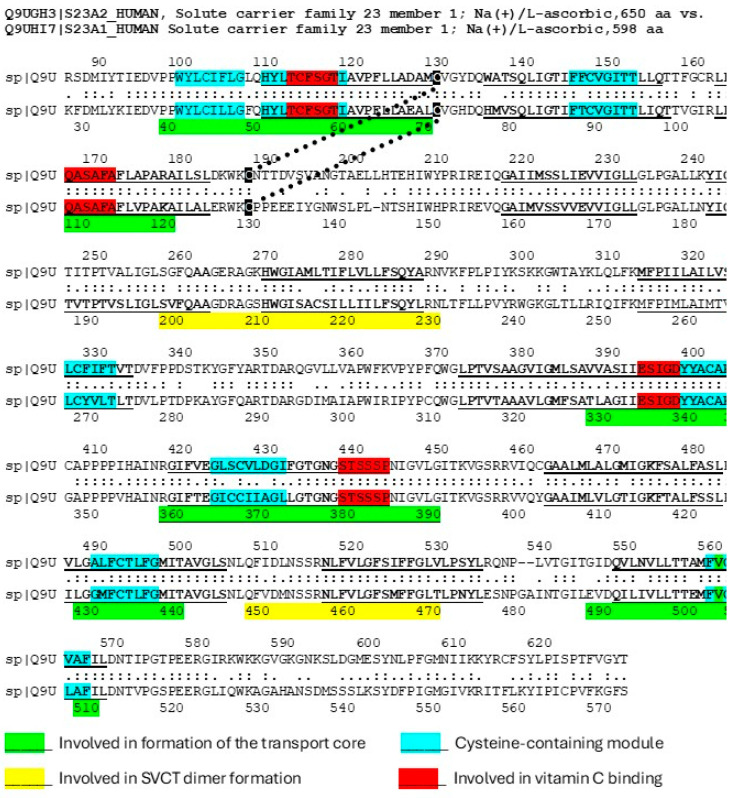
Sequences of human sodium-dependent vitamin C transporters type 1 and 2 showing the regions (green) involved in forming the transport core of the protein; the placement of cysteine-containing modules that mimic glutathione (blue); and sequences from several transmembrane regions that interact with region 110–120, which forms the primary vitamin C binding site during transport (red). Both the glutathione-like (blue) and associated transmembrane regions (red) appear to be modular. Sequences are from [134] while structural and binding information is from [135,136]. Underlined regions represent transmembrane regions of the transporter.

In the context of understanding the origins of interactomes involving transmembrane transporters, it is interesting to note that the uptake of vitamin C (ascorbic acid) is mediated by two mechanisms, one being sodium-dependent vitamin C transporters (SVCTs). The SVCT structure is clearly modular, integrating at least eight cysteine-containing sequences into its transport core, with several of these mimicking glutathione (Figure 5). Thus, MCMs appear to have been a selective force in ascorbate transport as in glucose transport.

The other vitamin C transport mechanism, which is operational in cells that lack SVCT, utilizes the oxidized form of ascorbate, dehydroascorbic acid (DHA), via glucose transporters in competition with glucose [137,138,139,140,141]. Once inside cells, glutathione converts the DHA back into ascorbate. However, under hyperglycemic conditions, the glucose–dehydroascorbate competition for glucose transporters results in tissue-specific deficiencies in ascorbate along with a corresponding decrease in glutathione production [137,142,143]. This glucose transporter mechanism for ascorbate transport reveals another aspect of MCMs as well. The structural similarities between dehydroascorbic acid and glucose are close enough that the concentration of ascorbic acid (and thus DHA) present in a sample alters the readings provided by glucose measuring devices based on glucose oxidases [144]. Vitamin C, through a variety of mechanisms including direct binding to glucose binding sites on proteins, also prevents non-enzymatic glycation of proteins including insulin [145,146,147]. Thus, vitamin C modulates glucose metabolism in multiple ways and glucose concentrations modulate ascorbic acid uptake and metabolism.

**Figure 6:**
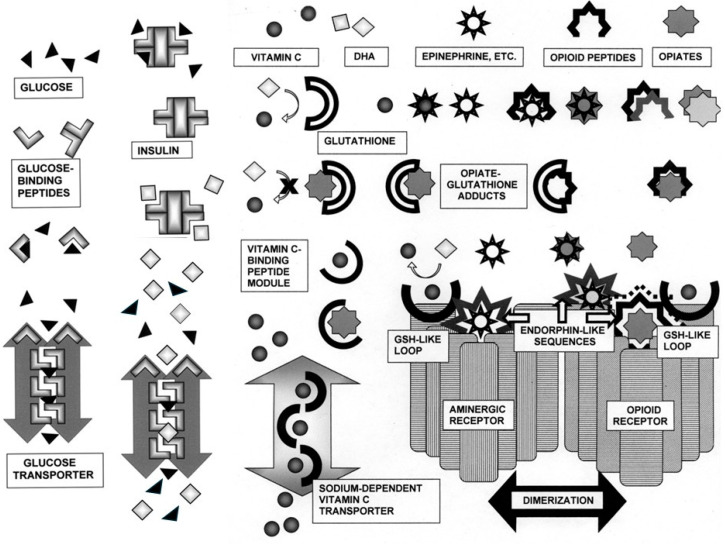
Schematic diagram of interactions among the small molecules glucose, vitamin C (ascorbic acid), dehydroascorbic acid (DHA), epinephrine and other catecholamines, opioid peptides such as enkephalins and endorphins, and opiates such as morphine. Many types of molecular complementarity are expressed in these interactions. The opioids and opiates self-aggregate; catecholamine bind to opiates and opioids and are co-stored and co-released in neurons and the adrenal gland; glucose binds to insulin and other structurally related glucose-binding peptides, mimics of which make up the transport core of glucose transporters. DHA, in turn, mimics glucose, utilizing glucose transporters to enter cells in competition with glucose, the latter antagonizing glutathione (GSH) activity and expression. Sodium-dependent vitamin C transporters transport ascorbic acid by means of glutathione-like modules in its transport core, and may possibly be antagonized by opiates. Opiates and opioids also block glutathione activity and the opiates can form covalent adducts with glutathione molecules that are excreted. Many of these complementary modules have apparently been integrated into aminergic and opioid receptors as well, where the ligand binding sites are made up of pro-enkephalin and endorphin-like sequences, and binding is enhanced by vitamin C binding to extracellular loop regions that mimic glutathione. In short, modules defined by small molecular complementarity have been utilized by evolution to network a wide range of metabolic functions. See text for additional details and references.

Finally, it is notable that, due to the mimicry of opiates and ascorbic acid (or more particularly DHA) simple prebiotic chemistry intrudes once again into this system of interactions in the form of the production of glutathione–opiate adducts, one of the mechanisms by which the body metabolizes opiates [148,149,150]. The production of these adducts unfortunately also dysregulates vitamin C metabolism by depleting glutathione, thereby having systemic effects through inhibition of its primary antioxidant function, as well as by binding covalently to other proteins exhibiting glutathione-like sequences [148], which may include aminergic receptors. The covalent reaction occurs spontaneously in vitro [151] and can be carried out by gut microbes [152], but mainly appears to be catalyzed in human beings and other mammals in the liver by the detoxifying enzyme cytochrome P450 [150,153]. Thus, the ascorbate–glutathione system integrates into the glucose–insulin–insulin receptor system [88] as well as into the adrenergic–opioid regulatory system [125,126] linking all three into an extended interactome (Figure 6).

Notably, all of the interactions described above, and their metabolic and systemic effects, are predictable from the network of small molecule interactions exhibited by glucose, ascorbic acid (or dehydroascorbic acid), adrenergic compounds and opioids with each other and with sugar-binding peptide modules (e.g., insulin), opioid peptides and glutathione. Thus, as in the examples summarized above, one can once again trace the origins of these modern, complex interactomes to evolution from highly conserved, simple prebiotic instances of molecularly complementary modules (MCMs).

## 4. PEI Evolution Case Study 2: Co-Evolution of Proteins and RNAs in the Origins of the Ribosome

The examples provided above hide an important mystery, which is how the molecularly complementary modularity that results in ligand–receptor, ligand–transporter, and enzyme–substrate pairings is integrated at the genetic level. Dwyer [57,58] has proposed that this process is mediated by mobile exons (“transposable exons” or “trexons”) that serve as modules that are ligated together to form novel gene products, but that mechanism assumes an already functioning transcription and translation system that can respond to selection on variations of metabolic systems. Thus, an examination of the evolution of the ribosome, which exists at the hub of the gene-to-protein interactome, may provide important clues as to how such modular exons may have arisen and resulted in highly integrated interactomes. This process requires feedback from the protein products of these exons to the exons themselves in order to implement selection for MCMs so that RNA–amino acid or RNA–peptide interactions were very likely among the foundational aspects of the origins of the transcription–translation system [154,155,156,157,158,159].

An explicit assumption of the discussion that follows is therefore that there was never an RNA world in which amino acids and peptides did not co-exist and play essential functional and structural roles. The rationale for this assumption exists in part because of the ease with which amino acids and peptides (see section above concerning glutathione synthesis) occur in even the simplest Miller-like prebiotic experiments [101,102,103], as do sugars such as glucose and ribose [103,160,161]. It is difficult to imagine a prebiotic chemical ecosystem in which only RNA was made, but not the amino acids and lipids and from which a translation system evolves organically [162,163,164]. On the other hand, the synthesis of RNA in aqueous solutions such as those that promote amino acid, peptide and sugar formation has generally been considered to be antithetical, or at best a serious challenge to the simultaneous synthesis of ribonucleotides (e.g., [165,166]). However, several plausible prebiotic pathways to the synthesis of nucleic acids in aqueous environments have recently been reported, most involving the use of catalytic surfaces such as clays or minerals and thus involving complementary molecular interactions as part of their mechanisms [167,168,169,170]. Physiologists, moreover, have long observed that cells have solved the aqueous solution synthesis of ribonucleotides and their polymers by stabilizing them with high concentrations of magnesium [171,172]. Recent Miller-like experiments by Root-Bernstein, et al. [103] provide evidence that nucleic acid bases, ribose and even adenine triphosphate (ATP) can be produced in aqueous solutions along with amino acids and peptides in the presence of magnesium sulphate, calcium phosphate as well as amino acids and peptides. Additionally, aptamer–amino acid and aptamer–peptide complexes have been characterized that mutually stabilize the components, thereby increasing fitness and arguing for the co-evolution of peptides and RNAs [173,174,175].

If, then, genes did not come first and give rise to the rest of the molecular components required for the emergence of cellular life, what kind of molecular entity might have kickstarted cellular life? While the RNA world hypothesis certainly dominates current discussions of origins of life problems (e.g., [176,177,178,179]), it should be evident that genes have no function without an entire transcription and translation system (and thus cells) to implement the information they carry. If genetic sequences were selected independent of any gene products that they might be able to encode, this raises severe problems for understanding how receptor–ligand, transporter–ligand and enzyme–substrate pairings could have evolved (see discussions above). There is no a priori reason to believe that selection for a self-replicating network of RNAs should or could serendipitously encode genes capable of generating a functional protein interactome. Moreover, due to the Levinthal paradox of the interactome [41] the probability is infinitely small that such a pre-existing RNA network could functionally give rise to the integrally networked cellular machinery necessary to translate such genes, even if they had spontaneously arisen. The number of possible permutations between even a small number of genes (a few hundred to a few thousand) and all of the possible peptides and proteins that could be randomly generated (trillions upon trillions) would be astronomical. The only way to evolve integrated networks is to limit their production from the outset through modular complementarity [44,50,180,181,182].

M. Root-Bernstein therefore proposed that perhaps genes did not come first, but rather something that could simultaneously transcribe genetic information, translate it and use the products to self-replicate. Could, she asked, the ribosome be the primordial entity that resulted from selection for modular complementarity selected to integrate genetic, transcriptional and translational functions simultaneously by bootstrapping nucleic acid–peptide modules?

The immediate implications were literally revolutionary. Modern dogma holds that ribosomal RNA (rRNA) holds no genetic information, playing a merely structural role within the ribosome framework and providing the catalytic center for ligating amino acids together into protein sequences. This translation function requires transfer RNAs (tRNAs) to mediate between the genetic information carried in messenger RNAs (mRNAs) that are specific to each protein sequence. So, if M. Root-Bernstein was correct, rRNA would have to encode all of the required tRNAs.

Furthermore, translation requires tRNA synthetase proteins to catalyze the addition of the appropriate amino acids to each tRNA. Thus, in order for the ribosome to be a self-replicating, primordial translation system, rRNA would have to encode genes that could be transcribed into mRNAs for the key proteins (or at least their active site modules) required for self-replication and translation. The most important of these proteins would likely be RNases, key ribosome-binding proteins (RBPs) and tRNA synthetases. Since rRNA, mRNA and tRNA are universally treated in textbooks as separate types of molecules without any known relationships, the predictions made from M. Root-Bernstein’s proposal appeared to be extremely unlikely, but they were testable and I agreed to help test the idea.

Long story short, we have published a series of highly cited papers demonstrating all of the points just enumerated. *E. coli* rRNA encodes all of the tRNAs in duplicate copies with between 60 and 80% fidelity with the modern tRNA sequences and which are predicted to fold into the typical tRNA cloverleaf structures (Root-Bernstein & Root-Bernstein, 2015 [183]). The rRNA also encodes the key functional modules of tRNA synthetases, RNases, and other polymerases that would be required to optimize RNA replication and translation. Thus, significant sequences of rRNAs mimic the mRNAs of key replication, transcription and translation proteins [183]. Moreover, the rRNAs also encode significant portions of key ribosomal binding proteins that encapsulate the rRNA, thereby providing the information required to reproduce its own structure [183], including, for example, the RNA-dependent RNA polymerases (RdRp) [184]. Even more surprisingly, the vast majority of these ribosomal binding proteins are known to bind not only to rRNA sequences, forming the mature ribosomal unit, but also bind to their own mRNA sequences (reviewed in [185]). In other words, ribosome-associated RNA sequences are molecularly complementary to the proteins they encode, not just through the translation of linear sequences but by being able to bind directly to each other. Moreover, the rRNA sequences to which these proteins bind are cognates of the mRNA sequences encoding the proteins, making the rRNA and mRNA sequences homologous [185]. In short, rRNA appears to once have had tRNA and mRNA functions as well, the ultimate in pleiofunctionality.

Modularity is also evident throughout the structure of the ribosome. rRNA sequences appear to be compounded from specific sequences of tRNA modules. This feature was first observed by Bloch and his colleagues [186,187,188,189,190] and has subsequently been verified using different techniques by Root-Bernstein & Root-Bernstein [183] and Demongeot and Seligmann [191,192,193]. The peptidyl translation core (PTC) appears to be composed of a concatenation of two or more of these tRNA-like sequences [194,195,196,197,198,199,200,201,202], which were likely complementary to each other, providing an intrinsic way to self-replicate [203,204]. This hypothesis has recently received experimental verification by several groups [205,206,207,208], one of which notably observed that proto-ribosome function required interplay with complementary peptides in order to stabilize and compartmentalize function [209]. Thus, peptide synthesis likely involved modular tRNA sequences that were self-complementary and stabilized by complementary peptides that were self-encoded. In other words, the proto-ribosome may have resembled a virus-like structure capable of self-replication, a capability exhibited by some ribozymes under artificial selection conditions [210,211,212].

Additionally, full-length tRNAs themselves appear to be composed of smaller, highly conserved stem-loop modules of between 22 and 31 nucleotides in length that were subsequently ligated to form full-length tRNAs [200,207,213,214,215,216]. Villarreal and Witzany [217,218] have presented evidence that the same type of stem-loop RNA sequences that are implicated in the origins of tRNAs (and thus the rest of ribosomal RNA formation) also created a diverse ecology or consortium of quasi-species that provided the foundation of prebiotic RNA networking more broadly [219], a point that will be taken up in a somewhat different context below. Their results are fully compatible with the account of ribosome formation suggested here. Thus, the ribosome exhibits multiple layers of modular construction in which tRNAs evolved to provide the structural and functional basis of rRNAs and rRNAs probably functioned simultaneously as structural material (rRNA), genetic information (rRNA) and messenger RNA (mRNA), while mediating the production of translation mediators (tRNA). These RNA sequences encode key protein modules that function as ribosomal proteins and enzymes and these proteins bind to rRNAs and mRNAs through molecular complementarity. The information required to perform all of these functions is encoded in overlapping reading frames, probably in both directions [183], a feature that is still utilized in some viruses and bacteria [220,221,222,223,224,225,226] and which has recently been observed in plants as well [227]—a possible example of persistence of an early, adaptive interactome.

**Figure 7:**
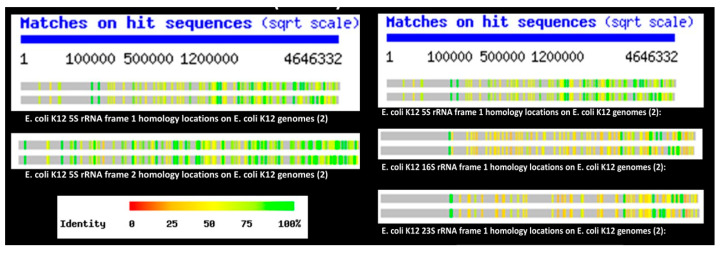
BLAST results showing locations and degree of homology between *E. coli* rRNA and the entire *E. coli* genome. Left: Two reading frames of 5S rRNA compared with two homologs of *E. coli* K12. Right: The 5S, 16S and 23S frame 1 homologies, each with two homologs of *E. coli* K12. The bright green lines indicate near 100% homology. These results suggest that the *E. coli* genome may have been constructed to a significant degree by multiplying ribosomal genes. Since these ribosomal genes contain tRNA and ribosomal protein-like sequences, the genome (and its products) as a whole would have been integrated intrinsically into the transcription and translation interactome from the outset.

**Figure 8:**
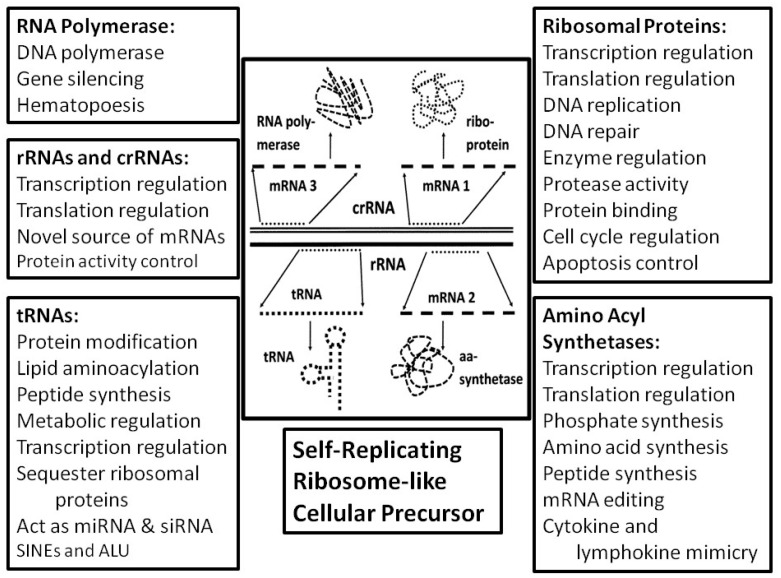
Summary of the non-ribosomal functions that have been discovered for ribosomal proteins, suggesting that the ribosome has played a central role in the development of the broader cellular interactome. Adapted from [228].

The final piece of this rethinking of ribosome structure/function is the fact that tRNA and rRNA-like sequences are encoded so frequently in bacterial chromosomes such as that of *E. coli* that they make up over 20% of the whole (Figure 7)—this represents far more copies of tRNA- and rRNA-like sequences than are required even for redundancy [228,229,230]—suggesting that tRNA and rRNA served as genetic modules from which higher order genetic constructs such as chromosomes, containing novel genes and proteins, were subsequently generated by sequence duplication and mutation. Indeed, ribosomal binding proteins have an extraordinary range of non-ribosomal functions (Figure 8) (reviewed in Root-Bernstein & Root-Bernstein, 2019 [228]), making them excellent candidates for the evolution of many functions unrelated to ribosome function, and tRNAs and rRNA fragments themselves play many pleiofunctional roles in modern cells. rRNA fragments, for example, mediate translation regulation and protein functions and can act as mRNA transcripts (reviewed in [228]). tRNAs play roles in lipid acylation, peptidoglycan modification, antibiotic synthesis, metal chelation, transcription regulation, control of outer membrane formation and binding of proteins to the ribosome (reviewed in [228]). In short, the ribosome appears to have provided the genetic and metabolic basis for an unexpectedly wide range of other cellular functions, all of these intrinsically integrated epistatically due to the extraordinary degree of modular complementarity present within the ribosome itself. The suggestion by Villarreal and Witzany [217,218] that quasi-species of stem-loop RNA more generally are at the heart of genetic organization may provide a broader understanding of genome structure beyond just rRNA-like sequences.

Understanding the evolution of the ribosome brings us back to Dwyer’s proposition that MCM formation was probably mediated by transposable exons (trexons) [57,58], thus linking the Case Studies above with the ribosome case study. The connection can be made through transposable elements such as SINEs and LINEs. SINEs are short interspersed nuclear elements, while LINEs are long interspersed nuclear elements. Both are highly abundant within modern genomes, ranging from endogenous retroviruses and microorganisms to mammals and are derived from tRNA-like and rRNA-like sequences [228,231,232,233,234]. As would be expected from the modularity of tRNAs and rRNAs, SINEs and LINEs have also been found to share common, modular bases [235,236,237]. Thus, the ribosome itself may have provided the genetic machinery by which MCMs were first encoded and then dispersed and varied throughout the genome by means of the transposable elements from which they were formed.

One possible logical conclusion that can be drawn from the data just described is that the ribosome is, in a sense, a giant virus around which the first modern cells evolved. From this perspective, one might conjecture that the ribosome, perhaps, evolved from an aggregation of networked, smaller, virus-like particles which, like modern viruses, were composed of RNA cores encoding the key proteins required to translate their genetic information into stable protein–RNA complexes. In this case, Villarreal and Witzany’s [217,218] RNA quasi-species may have been selected for encoding peptide or protein sequences capable of self-aggregating to their RNAs, thereby selecting for the very transposable elements that eventually encoded the ribosome itself. Such a scenario would explain how the ribosome evolved its own, extraordinarily information rich, integrated interactome. The same reasoning leads to the tentative conclusion that a gene–protein interactome may have been present from the very origins of replication and translation

The probability of finding all of this information packed into the rRNA sequences of ribosomes purely by chance is negligibly small, strongly arguing for a lengthy evolution selecting for the condensing and integration of multiple types of genetic, structural and functional information prior to the emergence of the last universal common ancestor. Thus, evolution appears to have selected for RNA sequences that encoded proteins capable of binding to their own generative sequences, thereby protecting both the RNA and the protein through the resulting complexation [238]. One prediction that we have not yet tested would therefore be that since rRNAs appear to be modular constructions derived from tRNA sequences, the tRNA sequences themselves should encode (if translated as mRNAs) self-binding protein modules. In other words, ribosomes may have evolved from compositionally diverse virus-like particles working in aggregate as an ecosystem capable of replication and translation—or what has been called a “virus world” [239,240,241,242].

**Figure 9:**
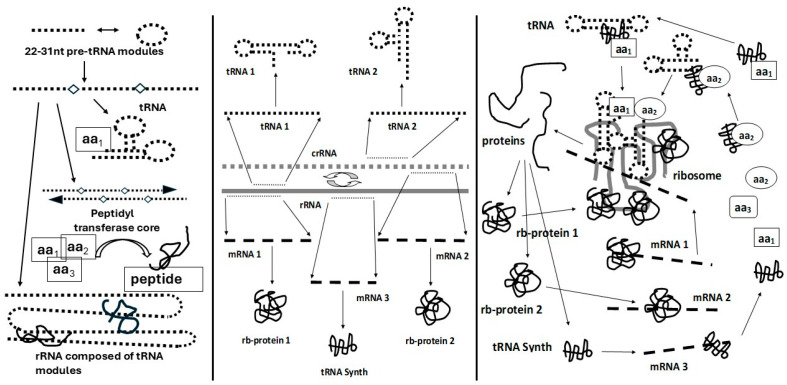
Schematic diagram illustrating the evolution of the ribosome from molecularly complementary modules, proceeding left-to-right. Studies suggest that tRNAs evolved from smaller RNA modules of 22 to 31 nucleotides (nt) in length that were ligated (diamonds). Complementary pairs of tRNAs form the peptidyl transferase core and may have been self-replicating. Some of these sequences may have encoded peptides that acted as RNA polymerases, synthetases and primitive amino acylating enzymes adding amino acids to tRNAs, thereby creating a self-replicating entity. Ligation of further tRNA-like modules expanded the rRNA and modern rRNAs contain all 20 types of tRNAs in multiple copies so that the rRNA may once have been able to make its own translation machinery. Additionally, rRNA contained “genes” that encoded peptide or protein modules with ribosome-related functions. Modern ribosome-binding protein (RBP)-like genes sequences are therefore found in the rRNA of modern organisms and their mRNAs mimic these rRNA sequences. Moreover, RBPs bind to both their own mRNAs and to similar sequences in rRNA. Thus, the ribosome contained rRNA that functioned not only as a structural element but also as mRNAs and tRNAs to encode the RBP modules required for ribosomal function. The primitive ribosome may therefore have had a structure like a giant virus and have been capable of reproducing itself. A gene–protein interactome may therefore have been present from the very origins of replication and translation. See text for additional details and references.

Thus, molecularly complementary modules can be traced from the precursors of tRNAs through rRNA to the genome itself and through the PTC to translation and the interactome that produced self-replicating ribosomal structures from which the modern translation system evolved (Figure 9). Complementarity between protein (or peptide) modules and RNAs and between proteins with other proteins then selected for the integral interactome that is the ribosome–the very center and progenitor, perhaps, of all cellular interactomes: MCMs leading to PEIs incarnate.

## 5. Conclusions: Why Selection for Systems Makes a Difference to How We Do Biology

Functions are always the result of specific interactions. No molecule (no machine part, for that matter) has a function on its own. DNA does not, intrinsically, carry genetic information. Its information capacity exists only in the context of a cell and the transcriptional and translational machinery that makes use of its sequence to make a protein. Lithium is not intrinsically an antidepressant (or lithium batteries are very happy, indeed!) but functions as a pharmaceutical only in particular contexts. While such reasoning may seem elementary or even simplistic, it cuts to the heart of what methods must be used in order to address the evolution of functions, functionality being the only trait upon which natural selection can operate. My contention is that functional traits can only be understood, and their origins revealed, by exploring sets of molecular interactions. The pieces of the puzzle of life contain the whole picture but do not explain why they are the only pieces that have been selected nor do they provide instructions for how to assemble them. Only their complementarity provides the necessary clues. Thus, this paper, and other papers cited in this article, proposes that MCM must be involved in that assembly process and PEI must be one necessary result.

Two general methodological implications follow from the MCM–PEI relationship that should apply to understanding the origins and evolution of any cellular system, and thereby provide tests of the overall proposition. One is that modern molecular systems should retain in their structures and interactions traces of their evolutionary histories that can be revealed through a “molecular paleontology” [48,52,53] similar to that used to track the morphological and genetic evolution of organisms. In practice, this assumption means that there should be patterns embedded within the structural components of molecular systems and their interactions that are not random but rather the results of the earliest forms of prebiotic selection for complementary modules.

Modularity, however, poses the challenge of how to use existing genomic and proteonomic tools for interrogating biological databases. Most similarity search programs have been optimized to explore global similarities that identify evolutionary tree types of relationships, not small modular ones that explore how evolution may have cobbled together bits and pieces to make assemblages. Thus, all of the genomic and proteonomic studies cited here have had to use innovative means to search for such modules by, for example, starting with very short sequences (which unfortunately are very likely to generate many), mostly artefactual resemblances, and then using sophisticated statistical methods to evaluate the results. Additionally, existing search engines are not able to identify complementary sequences among peptides and proteins at all, or between peptides or proteins and DNA or RNA sequences, except by homology to certain motifs previously discovered experimentally. No programmatic tool exists for predicting small molecular complementarity at all, nor are there databases of such interactions available at the present time. Thus, many of the kinds of studies that are needed for extrapolation will require the development of new tools and many additional experiments.

The second methodological implication is that traits are the expressions of systems of molecular (or cellular) interactions, and therefore not merely the result of variations on individual genes, proteins or other molecules. Epistasis and complementarity are indissolubly linked. It also follows that living systems are non-decomposable into their molecular units because an inherent property of such systems is the sets of molecular complementarities that make possible their organization. Stated another way, function results from the formation of interactomes. Thus, my hypothesis predicts that living systems are the products of hierarchies of subsystems selected on the basis of MCM for PEI. To study such systems requires experimenters to abandon testing the effects of one molecule at a time and instead begin to explore more explicitly and with more sophisticated experiments, how systems respond to pairs or higher-order mixtures of molecules. Every experiment cited in this paper has been of this multi-component type, a type that is not at all common in molecular biology at present.

This second methodological consideration yields further testable implications that yield specific experimental predictions: as suggested by the examples provided here, molecules that are biologically active and modify the activity of other bioactive molecules are likely to be molecularly complementary; and conversely, when two biologically active molecules are molecularly complementary, they are likely to alter each other’s physiological activity [44,51]. These complementary principles follow directly from the MCM mechanism of interactome formation and the retention of core MCMs within modern interactomes. It is not by chance that it is therefore possible to identify and explain drug interactions (both useful and harmful) using these complementary principles, since the interactivity is built into the system [51,71].

In short, this and the other papers cited here present what is being called a “third way” of thinking about evolution [243,244,245,246] which stands somewhere between mechanistic reductionism, which asserts that detailed knowledge of the components of a system can define the functions of a system, and wholism, which asserts that life is not reducible to its components. As noted in the introduction, Jacob called this intermediate approach “evolutionary bricolage” [1,2], a concept recently applied explicitly, and in a manner consistent with the approach used here, to the origins of the translation system by Seelig and Chen [247]. MCM is also based upon a “bricolage” approach to originating self-organizing interactomes and assumes a hierarchical systems approach which proposes that the functions characterizing life can only be understood by identifying the non-random interactions of the components of the systems that yield emergent properties, which are unpredictable from the properties of the individual components. These systems are, according to this “third way”, organized in hierarchies, each of which then yields additional novel emergent properties, finally yielding the functional whole that we know as a living cell. Thus, knowledge of the components of each system or subsystem is necessary to implement this “third way”, but it is not sufficient because the individual components cannot predict the pleiofunctionality that characterizes the several or many roles each component plays within a cell. Similarly, an understanding of the structural and functional characteristics of the living whole is also necessary, since the purpose of such studies is to explain the ultimate functions of the living cell, but can only be understood in terms of the hierarchy of epistatic interactomes represented by the hierarchies of subsystems composing the whole. Thus, only by identifying the constraints that yield such fit systems and subsystems and examining how their components interact to yield novel functions can reductionist and wholist thinking be bridged to yield a stepwise story of how evolution by natural selection yielded living interactomes cobbled together from a surprisingly limited range of pleiofunctional components. MCM was (and remains) one of these selection pressures. Whether there are others involved in interactome formation and retention remains to be seen.

The test of these general propositions will, of course, be to investigate and explain the origins of specific instances of interactomes other than the ones reviewed here. Many possibilities suggest themselves and only two will be mentioned here. One involves the possible MCM origins of the many links between tryptophan, serotonin and other indoleamines with coenzymes such a riboflavin and thiamine. Serotonin and tryptophan forms charge transfer complexes with both riboflavin and thiamine [248,249,250,251]; riboflavin acts as a cofactor for monoamine oxidases [252], is an essential coenzyme in the synthetic pathway of indolamines and in their protection against oxidation [253] and has been found to synergize with serotonin-based treatments to prevent migraines [254,255]. Thus, as in the ascorbate–glutathione system described above, it is possible that indolemine–flavin complementarity was the basis for the system of metabolic and physiological interactions that subsequently evolved. A second, and more general area for future research may involve biomolecular condensates. Biomolecular condensates are dynamic, membraneless droplets inside cells that concentrate specific proteins, RNA and DNA to organize cellular functions like transcription, ribosome biogenesis, stress response and signal transduction, acting as crucial compartments for biochemical reactions without needing a surrounding membrane and often forming via liquid–liquid phase separation. Such condensates are always composed of multiple types of molecules and organized phase separation is driven by a combination of molecular concentrations and complementary interactions [256,257,258]. To what extent the resulting self-organization results specifically from evolutionarily conserved complementary modules is unknown at present and might provide an interesting test of the MCM to PEI hypothesis developed here.

In concluding, I want to quote two groups that have been working along parallel lines to my own and have reached similar conclusions about the roles of molecular complementarity in interactome formation based on general theoretical considerations and on very different sets of examples than the ones presented here. One group is led by Peter Csermely, whose book *Weak Links: Stabilizers of Complex Systems from Proteins to Social Networks* lays out the importance of molecular (and other forms of) complementarity as essential mechanisms of interactome formation: “Weak links came as a great surprise to me. It was not so much their existence, which is obvious, but their importance that was the new message from my reading and which constitutes the main message of this book. These links form most of the links inside and around us … Weak links stabilize all complex systems. Weak links give us a universal key to understanding network diversity and stability, and they are the major actors….” ([43] pp. xi & 275). More broadly, Caetano-Anollés et al. have recently written: “Networks describe how parts associate with each other to form integrated systems which often have modular and hierarchical structure. In biology, network growth involves two processes, one that unifies and the other that diversifies… In the first phase, parts are at first weakly linked and associate variously. As they diversify, they compete with each other and are often selected for performance. The emerging interactions constrain their structure and associations. This causes parts to self-organize into modules with tight linkage. In the second phase, variants of the modules diversify and become new parts for a new generative cycle of higher level organization. The paradigm predicts the rise of hierarchical modularity in evolving networks at different timescales and complexity levels” [42]. When multiple groups arrive as similar conclusions from diverse starting points, there is often something worth pursuing at the intersection.

## Data Availability

No new data were created or analyzed in this study.
